# Repressor Dimerization in the Zebrafish Somitogenesis Clock

**DOI:** 10.1371/journal.pcbi.0030032

**Published:** 2007-02-16

**Authors:** Olivier Cinquin

**Affiliations:** 1 Centre for Mathematics and Physics in the Life Sciences and Experimental Biology, University College London, London, United Kingdom; 2 Department of Anatomy and Developmental Biology, University College London, London, United Kingdom; 3 Laboratoire TIMC-IMAG, Centre national de la recherche scientifique, Université Joseph Fourier, Faculté de Médecine, La Tronche, France; Lawrence Berkeley National Laboratory, United States of America

## Abstract

The oscillations of the somitogenesis clock are linked to the fundamental process of vertebrate embryo segmentation, yet little is known about their generation. In zebrafish, it has been proposed that Her proteins repress the transcription of their own mRNA. However, in its simplest form, this model is incompatible with the fact that morpholino knockdown of Her proteins can impair expression of their mRNA. Simple self-repression models also do not account for the spatiotemporal pattern of gene expression, with waves of gene expression shrinking as they propagate. Here we study computationally the networks generated by the wealth of dimerization possibilities amongst transcriptional repressors in the zebrafish somitogenesis clock. These networks can reproduce knockdown phenotypes, and strongly suggest the existence of a Her1–Her7 heterodimer, so far untested experimentally. The networks are the first reported to reproduce the spatiotemporal pattern of the zebrafish somitogenesis clock; they shed new light on the role of Her13.2, the only known link between the somitogenesis clock and positional information in the paraxial mesoderm. The networks can also account for perturbations of the clock by manipulation of FGF signaling. Achieving an understanding of the interplay between clock oscillations and positional information is a crucial first step in the investigation of the segmentation mechanism.

## Author Summary

Vertebrate embryos acquire a segmented structure along the anteroposterior axis. Segmentation is critical for patterning of other structures (such as nerves, vertebrae, muscles, and blood vessels) and occurs by the rhythmic separation of balls of cells, called somites, from the anterior end of their precursor tissue, called the presomitic mesoderm. These rhythmic events are associated with oscillatory gene expression in the presomitic mesoderm: waves of gene expression originate at the posterior end and spread anteriorly. When a wave reaches the anterior end, a pair of new somites detaches. The set of genes whose expression oscillates is termed the “somitogenesis clock.” Even though the zebrafish somitogenesis clock has been the subject of intensive study, it is not clear how its oscillations are generated. It has been proposed that the mechanism involves a simple negative feedback loop, with proteins of the Her family periodically repressing their own expression. However, this is incompatible with some experimental results and does not explain how the spatiotemporal pattern of gene expression is generated. Here I propose a model—based on physical interactions between Her proteins—that is compatible with experimental results, and that explains how positional information is used to generate the spatiotemporal pattern of gene expression.

## Introduction

A somitogenesis clock, linked to the vertebrate segmentation process, has been uncovered in mouse, chick, and zebrafish [1]. Genes involved show oscillatory expression in the presomitic mesoderm (PSM) and are mostly related to the Notch pathway in all three species. In zebrafish PSM, oscillatory genes include *her1* and *her7,* transcriptional repressors whose expression is thought to be enhanced by Notch signaling, and *deltaC,* which encodes a Notch ligand; expression of each of these three genes is necessary for correct oscillatory expression of all three [2,3]. The Her1 protein represses expression from its own promoter in a cell-culture assay [4], and it has been proposed that self-repression of *her* genes could drive the somitogenesis clock [5–7]. Intriguingly, however, morpholino blocking of *her1* or *her7* mRNA translation can lead to downregulation of their transcription [2], while a simple negative feedback loop would predict upregulation of transcription if the repressor protein cannot be translated. What is more, the significance of the requirement for Her13.2, a cofactor for Her self-repression [4], remains unexplored in mathematical models. Here we study computationally the properties of networks where Her proteins must dimerize to act as repressors. Networks are assessed both for compatibility with morpholino knockdown phenotypes and for correct reproduction of the collective creation of an oscillatory pattern in the PSM.

**Figure 1:**
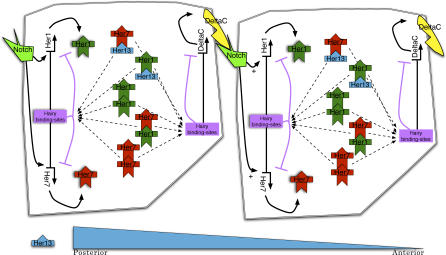
Model of the Interactions between Her Proteins in the Zebrafish Network Driving Somitogenesis Oscillations Her1, Her7, and Her13.2 (abbreviated to Her13) can form heterodimers, and Her1 and Her7 can also form homodimers. Each dimer species competes to set the level of repression of *her1, her7,* and *deltaC* to a specific level. (This is inspired by the experimental result that a combination of Her1 and Her13.2 leads to more potent repression than Her1 alone.) Intercellular communication occurs through DeltaC-Notch signaling, which enhances *her1*, *her7,* and *deltaC* transcription. *her13.2* mRNA is expressed in a posterior–anterior gradient in the PSM.

Her13.2 is expressed in a posterior–anterior gradient in zebrafish PSM and heterodimerizes with Her1 to enhance Her1 autorepression [4]. Dimerization is a common feature of the basic helix loop helix family [8], and in the following it is assumed that Her13.2 can heterodimerize with Her1 or Her7, that Her1 and Her7 can also homodimerize or heterodimerize with each other, and that the resulting dimers repress expression of *her1, her7,* and *deltaC*. *her1* and *her7* share a common 12-kb promoter [2], which contains nine copies of the consensus hairy binding site (CACGCG [9]). In the model proposed here, depicted in Figure 1, all dimer combinations between Her1, Her7, and Her13.2 compete for binding to these sites, and repress *her1* and *her7* transcription with strengths specific to each dimer combination. It is assumed that the *deltaC* promoter functions similarly to that of *her1* and *her7,* in agreement with closely overlapping expression patterns in the PSM [2,5,6,10,11]; this does not take into account some differences in expression patterns observed in *deltaC* mutants [3]. Delays are taken into account for mRNA transcription and export from the nucleus and for protein translation.

None of the biochemical reaction rates has been determined experimentally. To investigate the behavior of the model, we therefore sampled values from extended, biologically realistic ranges. We screened for suitable oscillations, with at least a 5-fold difference between mRNA peak and basal levels (see Methods for a full definition), and for reproduction of experimental results obtained by perturbation of the oscillatory machinery. Because mRNA and protein copy numbers often reach low absolute levels at the trough of their oscillatory cycle, the effect of internal noise was assayed using stochastic simulations, with the same parameter sets that were identified as providing suitable deterministic oscillations. Possible reactions in the system were the same in the deterministic and stochastic cases, but stochastic simulations considered all individual reaction events explicitly.

## Results

### Mathematical Formulation of the Model

#### Single cells.

In the deterministic case, the framework used was that of delay differential equations, with variables representing a continuous approximation of mRNA and protein copy numbers in each cell. Binding and unbinding reactions between proteins were considered explicitly (rather than considering dimers to be at equilibrium), because—though they happen at a very high rate—it could not be excluded outright that the reactions could have had a detectable effect on the kinetics of oscillations (no such effect was directly observed, however). It was assumed that Her proteins are quickly and efficiently imported into the nucleus, and that dimerization occurs following the law of mass action, independent of DNA binding. (DNA can influence dimerization of bHLH proteins in vitro [12], but data about kinetics and equilibria of in vivo dimerization is missing.) To translate copy number to concentration, the diameter of nuclei was assumed to be 5 μm [7]. The function used to describe repression of transcription by Her proteins is a phenomenological one and does not explicitly take into account the binding of proteins to the promoters. (Protein–DNA binding reactions normally happen on an extremely fast time-scale [13]; see also [14] for an interesting discussion of the effects of the speeds of response of promoters.) Different dimer species are assumed to exert their activity on the promoter with a phenomenological cooperativity of three, which could stem from cooperative binding of each dimer species to Hairy sites in the promoter (a cooperativity of two leads to similar oscillations). Dimer species are assumed to compete for the establishment of a certain level of repression on the promoter (the more of dimer species X, the closer the repression level gets to that set by X, all other things being equal). Repressive activity is saturable, as suggested by experiments that show incomplete repression of reporters in situations where Her proteins should be in vast excess of the promoters they repress [4]. Finally, delays in transcription and export of mRNA as well as translation of proteins are taken into account for *her1, her7,* and *deltaC.* (This is unnecessary for *her13.2,* as this gene does not show oscillatory expression.)

For each cell, the equations are:

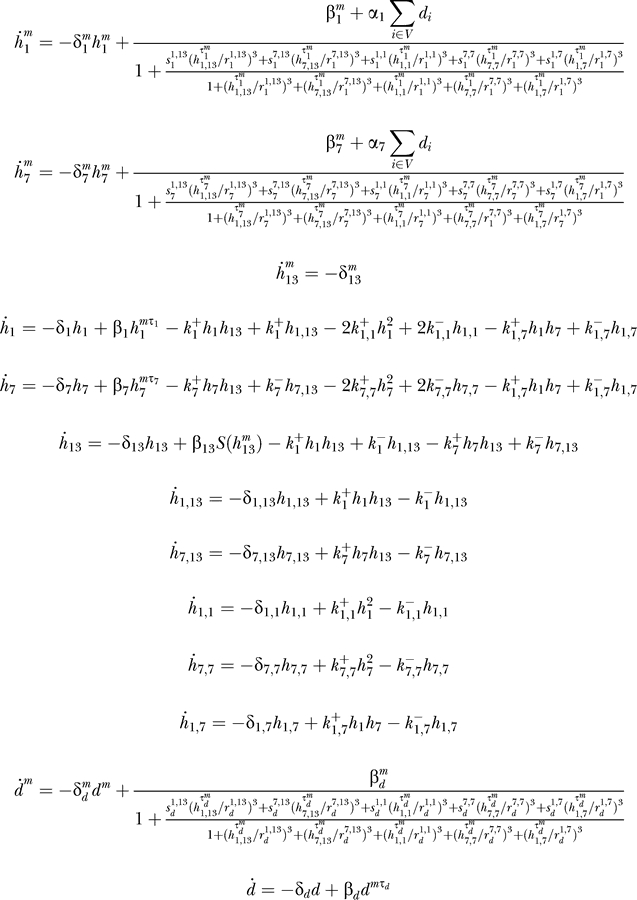
where 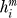 is the mRNA copy number of *her i* (*i* = 1, 7, or 13, the latter being an abbreviation of 13.2), *h_i_* is Her i protein copy number, and *d^m^* and *d* are copy numbers of *deltaC* mRNA and protein, respectively. 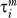 is an operator that shifts time values of the functions it is applied to by the delay corresponding to transcription, splicing, and export to the cytosol of *her i* (*i* = 1, 7) mRNA or *deltaC* mRNA (*i* = *d*). τ*_i_* is similar and is used for delays corresponding to protein translation. The term 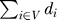 represents the sum of Delta protein copy numbers from neighboring cells (the two nearest neighbors on the left and right of each oscillator in the chain). *S* is a continuous approximation of a step function (see Methods).

Parameters other than delays are binding (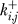 ) and unbinding (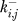 ) rates for dimer Her i–Her j, rates of exponential decay for mRNAs (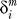 ) and protein monomers (*δ_i_*) and dimers (*δ_i,j_*), and maximal rates of synthesis for mRNAs (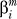 ) and proteins (*β_i_*).

The first term in each equation represents degradation of the corresponding species. Synthesis terms for *her1, her7,* and *deltaC* mRNAs depend on the amount of repression set by the combination of Her dimers. Dimerization terms appear as products of Her copy numbers (e.g., the rate of formation of the Her1–Her7 heterodimer is 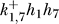 ), while unbinding events are proportional to the quantity of the corresponding species (e.g., the rate of splitting of the Her1–Her7 heterodimer is 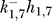 ).

#### Parameter ranges.

Most of the parameter ranges used for random sampling were selected to cover a wide range of realistic values. Degradation rates were chosen so that lifetimes were no less than 2 min. Maximal synthesis rates were 40 protein copies per mRNA per minute and 200 mRNA transcripts per minute. Protein dimerization rates ranged between 10^5^M^−1^s^−1^ and 10^7^M^−1^s^−1^, and affinities were chosen to lie between 0.1 nM and 10 nM. The range for delays in synthesis and mRNA nuclear export had a smaller lower bound than would be expected biologically, in order to study the influence of delays. The upper bound for transcriptional delays was also smaller than that used in [7]. This is because delays in mRNA maturation are implicitly assumed in [7] to stem from sequential mRNA transcription and intron removal, whereas splicing seems to be a cotranscriptional event [15,16], and the delays due to transcription elongation and mRNA splicing do not add up, because they probably happen in parallel. (Additionally, splicing reactions can happen cooperatively [17], and a delay due to splicing does not necessarily increase linearly with the number of introns.)

**Figure 2:**
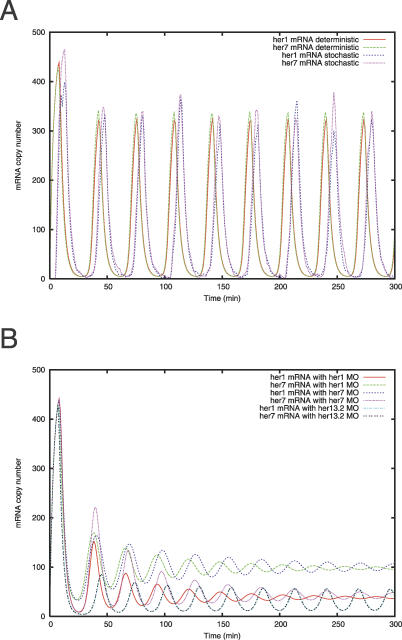
Numerical Simulations of the Model Sketched in Figure 1, without DeltaC (See Table 1 for Values of Parameters) Overlay of deterministic and stochastic simulation results (A) and deterministic simulation results with various simulated knockdowns (B).

**Figure 3:**
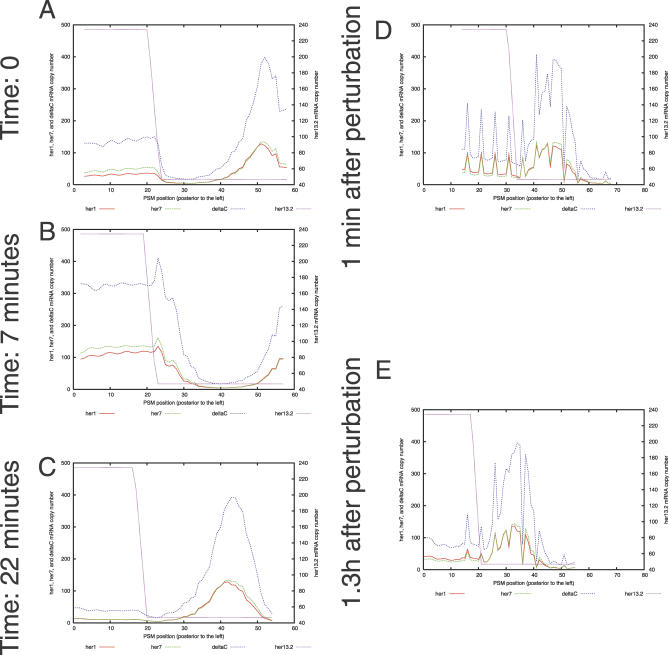
Deterministic Simulations of a Chain of Oscillators Corresponding to the Model Sketched in Figure 1 (See Table 1 for Values of Parameters) Waves of expression spread from posterior to anterior PSM (A–C), as observed experimentally. To test the effect of oscillator-coupling through DeltaC-Notch signaling, a perturbation was induced by delaying a number of oscillators in the chain by 5 min (D). After 1.3 h, the resulting perturbations in the spatial pattern were reduced (E); note that cells corresponding to indexes smaller than 13 on the *x*-axis should show no perturbation, because they were added to the chain after the initial perturbation.

**Figure 4:**
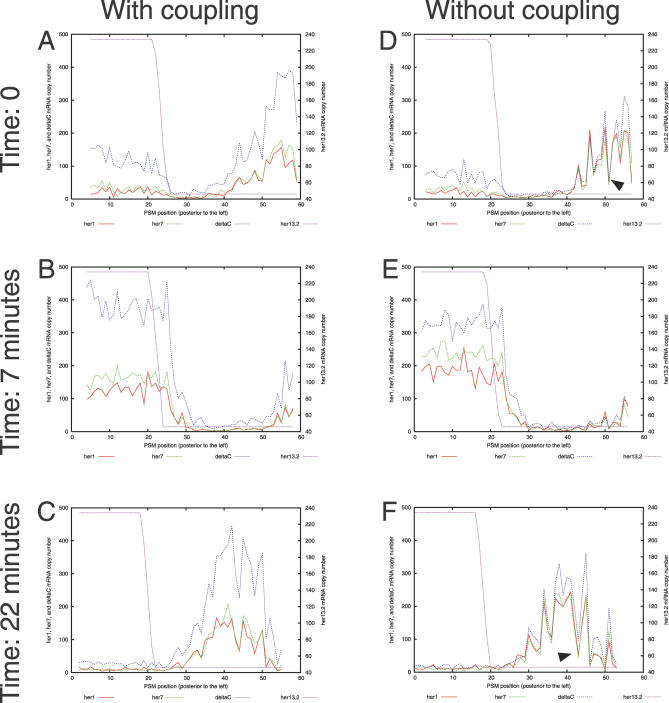
Stochastic Simulations of a Chain of Oscillators Corresponding to the Model Sketched in Figure 1, with Normal Coupling through Delta-Notch Signaling (A–C) and with Disrupted Coupling (D–F) When coupling was absent, some cells were noticeably out of synchrony with their neighbors (arrowheads), and local synchrony was not as strong. The transcription rate of *her1* and *her7* was increased in (D–F) to make up for the loss of Notch signaling.

**Figure 5:**
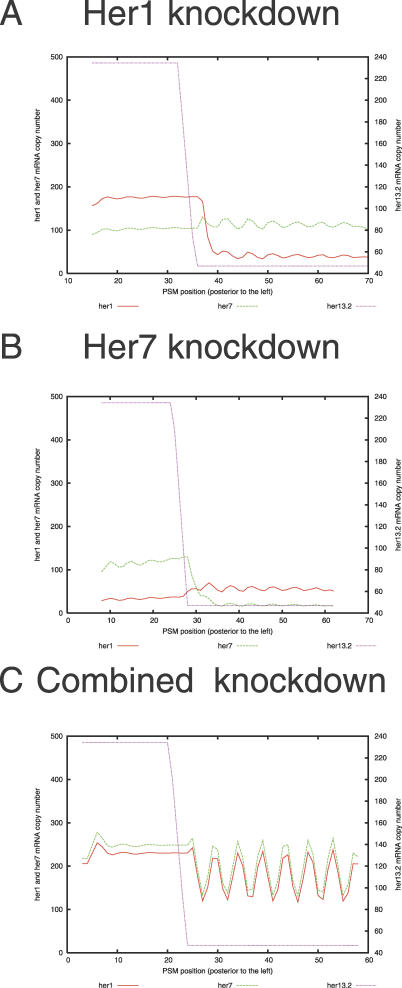
Simulated Translation Knockdown of *her1* (A), *her7* (B), and Combined *her1* and *her7* (C) For single knockdowns, residual oscillations of diminished relative amplitude were observed, and stripe formation in anterior PSM was disrupted. For the combined knockdown, *her1* and *her7* were upregulated and did not oscillate in posterior PSM, but did oscillate in a salt-and-pepper pattern in anterior PSM.

**Figure 6:**
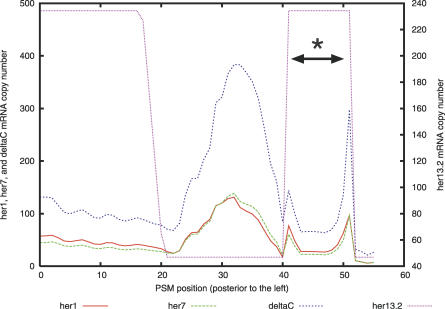
Simulated Grafting of an FGF-8 Coated Bead (Star) *her13.2* mRNA was assumed to be ectopically expressed, at the same level as in posterior PSM, in an area centered around the bead and marked by arrows.

Table 1 lists parameter ranges, as well as specific values for simulations shown in Figures 2–6. Some of these values fall outside of the ranges, because—after random selection—parameters were hand-tuned for reproduction of the spatiotemporal pattern. Parameters related to *her7* mRNA or protein and which do not appear in Table 1 were set in each simulation to the value of their *her1* counterpart, in order to reduce the dimensionality of the parameter set.

**Table 1:**
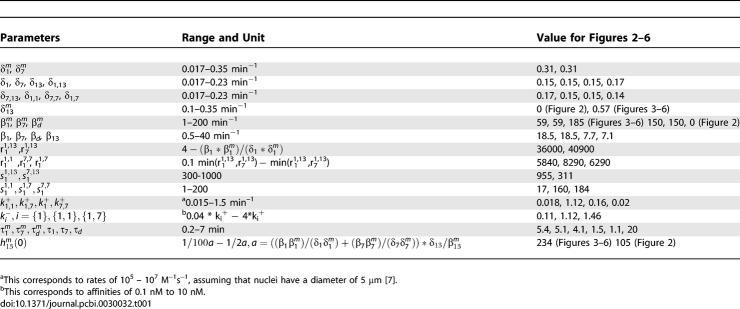
Ranges from Which Parameters Were Selected at Random for Numerical Simulations

#### Groups of cells.

The PSM is a three-dimensional (3-D) structure. A mediolateral spread of expression waves has been described in chick [18], but for the purposes of this study only the anteroposterior axis is of interest. The model structure was thus chosen to be a chain (comprising about 50 cells). Each cell influenced its two anterior and posterior neighbors.

#### Stochastic formulation.

The stochastic model was formulated as a direct translation of the delay differential equation model. All events (protein binding, unbinding, synthesis, and degradation) happened following an exponential law whose rate was the same as the reaction rate in the deterministic case. The only difference was that Her i homodimerization rates were proportional to *h_i_* (*h_i_* – 1), rather than 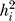 , as in the deterministic case. As in the deterministic case, events of Her dimers binding to the promoters or unbinding were not explicitly modeled. (Again, this is because the function describing transcriptional repression by Her dimers is of a phenomenological nature, and protein–DNA binding and unbinding events are expected to be very fast compared with other events being modeled.) Noise generated by the transcription and translation machineries [19,20], or stemming from mRNA processing and nuclear translocation, was not taken into account and remains the subject of further investigation.

### Oscillations in Single Cells and Knockdown Phenotypes

The model depicted in Figure 1 readily gives rise to single-cell oscillations with parameters sampled in the ranges detailed above. A parameter set was deemed robust if each parameter could be varied individually by 50% or more without disrupting oscillations. By this criterion, 50% of 300 randomly selected parameter sets were found to be robust.

As a first step to identify parameter sets reproducing the morpholino-induced disruption of the spatiotemporal pattern of oscillation described in [2], parameter sets were screened with single-cell simulations for downregulation of *her1* and *her7* mRNA when the Her1 or Her7 translation rates were divided by ten (corresponding to 90% morpholino efficiency in blocking protein translation). Such parameter sets occurred at a low frequency (see Figure 2 for a representative example). While general robustness of the model with these parameter sets was comparable with that in the general case (30% of parameter sets were found to be robust), the parameters with the strongest influence on the oscillation period were found to be strikingly different. Parameter sets leading to correct reproduction of the morpholino phenotypes also led to a very high sensitivity of the oscillation period to the degradation rate of the Her1–Her7 dimer (and to a lower extent to various parameters describing its repressive activity): for 30% of identified parameter sets, variation of the Her1–Her7 degradation rate yielded a higher variation in period than variation of any parameter not related to delays of transcription and translation. In the general case, period-sensitivity to individual parameter variation was more even across parameters and highest for the *her1* mRNA degradation rate. (For 10% of identified parameter sets, variation of that degradation rate led to the highest period variation amongst parameters other than delays.) This suggests a central role for a Her1–Her7 heterodimer, which can be tested experimentally.

Examination of the parameter values leading to correct reproduction of the *her1* and *her7* morpholino knockdown phenotypes showed that the repression strength of the Her1–Her7 heterodimer was strongly biased towards lower values than that of other repressive dimers. This suggests that the mechanism by which Her1 and Her7 are required for their own expression is by Her1–Her7 heterodimers acting as a “protective” species: Her1–Her7 heterodimers repress *her1* and *her7* expression, but compete with other dimer combinations that repress expression more strongly.

Parameter sets reproducing the *her13.2* knockdown phenotype (which consists of disrupted oscillations [4]) were identified independently of those reproducing the *her1* and *her7* knockdown phenotypes and did not show any notable difference in period distribution. Of the parameter sets leading to correct reproduction of *her1* and *her7* knockdown phenotypes, 10% also led to disruption of the oscillations when *her13.2* expression was knocked down (see example in Figure 2).

### Reproduction of the Full Spatiotemporal Pattern

We have so far addressed oscillations at the level of individual cells. One important feature of the somitogenesis clock is that waves of expression sweep from posterior PSM to anterior PSM and shrink in the process [10]. The spread of an intercellular signal is not necessary for the short-term maintenance of the oscillatory pattern in mouse and chick [21–23], but it has been suggested that cellular oscillators can influence their neighbors [11,24–26]. In the case of the chick and mouse somitogenesis clocks, a gradient in the strength of intercellular coupling can lead to the formation of the correct collective pattern of oscillatory expression [24]. In those species, no molecular link is currently known between the oscillatory machinery and positional information in the PSM. However, Her13.2 provides such a link in the zebrafish clock [4], which makes it possible to investigate the detail of the mechanism.

To assess whether graded expression of *her13.2* could prompt a linear chain of oscillators to form the correct collective pattern of oscillatory expression, a screen was carried out in which the dynamic structure of the PSM was reproduced by adding cells at regular intervals at the posterior end of the chain (corresponding to convergent extension and ingression from the tailbud [27]) and removing cells at the anterior end. (Cells were removed continuously, rather than in blocks corresponding to somites, because the process of segmentation, which is controlled by poorly understood molecular mechanisms, is not the subject of this study.) Each cell influenced its two anterior and posterior neighbors by providing them with the ligand Delta for Notch receptor activation, leading to increased *her* and *deltaC* transcription (the dynamics of Notch signaling were not modeled explicitly). Expression of *her13.2* mRNA was assumed to be high in posterior PSM and to drop sharply in anterior PSM [4]; the regulation of *her13.2* by FGF-8 was not modelled explicitly. The rates of *her13.2* mRNA decay and of cell addition at the anterior end set the length of the PSM, and therefore the number of oscillations each cell experienced while in the PSM (that number was set to 12, as can be estimated from [3,27,28]).

A number of parameter sets were identified for which *her* and *deltaC* waves of expression swept from posterior to anterior, their width becoming restricted as they went along (see Figure 3A–3C and Video S1 for a representative example). The number of stripes observable at any given time depends on parameter sets and spans the range observed experimentally (from one in old embryos at specific phases, to up to three in younger embryos [10]); it was not attempted to reproduce precisely the rate at which stripes decrease in length. Positional information provided by Her13.2, as identified experimentally, is therefore sufficient to arrange oscillatory expression in the PSM in the correct pattern, within the framework of the model proposed here.

To determine whether the role of Her13.2 is to modulate intercellular coupling or to act on individual oscillators, simulations were run with no intercellular coupling (this was performed by abolishing DeltaC translation, or by replacing Notch signaling with fictitious autocrine signaling). For all parameters studied, it was found that intercellular coupling could be removed without destroying the spatiotemporal pattern. For some parameter sets, this loss of Notch needed to be compensated by an increase in the transcription rates of *her1* and *her7*. A shift in oscillatory phase and a slight change in the oscillatory period occur when *her13.2* mRNA expression drops (unpublished data); this is sufficient to set up the spatiotemporal pattern. Note that this does not contradict the fact that Notch signaling is necessary for oscillations in vivo. The model studied here has parameter sets for which Notch signaling is required for individual cellular oscillations with the correct pattern (by providing a sufficient level of *her* expression) as well as for intercellular coupling. In addition, the model has parameter sets for which Notch is required for intercellular coupling only. The first set of parameters corresponds to the in vivo behavior of the oscillator (such a parameter set was used to produce Figures 2–6). In vivo, intercellular coupling through Notch signaling could have the additional role of setting up the very first waves of expression, which are not the object of this study.

### Oscillator Coupling and Noise

Even if intercellular coupling has no role in the establishment of the oscillatory pattern under the simplified conditions studied here, it could have a crucial role in synchronizing cells in vivo [11,29]. Perturbations were simulated by delaying a number of oscillators in the chain by 5 min. For some parameter sets, such perturbations were resorbed within a few rounds of oscillation (see Figure 3D–3E and Video S1 at 50 min for an example). Synchronization was much stronger for cells located in posterior PSM than for cells in anterior PSM. This might be the basis for greater plasticity of posterior PSM as compared with anterior PSM, as observed in chick [30].

To further study the role of intercellular coupling in resistance to noise, the behavior of chains of oscillators in the presence of molecular noise was assessed as described above for individual oscillators. Due to the high computational and memory costs of simulating individual reaction events with delays for a system comprising more than 50 oscillators with 13 variables each, only a very small number of parameter sets could be studied. Nonetheless, parameter sets were identified for which the patterns of oscillation in stochastic simulations were as expected, close to the deterministic form (Figure 4 and Video S2). For such parameter sets, closely similar successive rounds of oscillation showed low variability between stochastic realizations. A stochastic simulation was run with disrupted coupling, with the same parameter set as used in Figure 3. A few cells went noticeably out of synchrony with their neighbors, and local synchrony was generally not as strong as with coupling, but the global spatiotemporal pattern was not disrupted (Figure 4D–4F and Video S3). Thus, even though this parameter set leads to perturbation resorption in posterior PSM (Figure 3D–3E), the molecular clock is sufficiently precise that this capacity is not required to maintain the spatiotemporal pattern with the molecular noise simulated here.

### Disruption of the Spatiotemporal Pattern

Morpholino knockdown of *her1* leads to residual *her1* and *her7* oscillations in posterior PSM and to defective stripe formation in anterior PSM [2]. Knockdown of *her7* leads to expression of *her1* throughout the PSM (with no stripes) and to posterior expression of *her7* [2]. Detailed comparison of simulation data and experimental data is not possible because the latter is not quantitative, but most general features can be reproduced. Simulated knockdown of *her1* or *her7* on the parameter set used in Figure 3 showed residual oscillations in posterior PSM, with a high level of basal expression (see Figure 5A–5B and Video S4 for the *her1* knockdown, and Video S5 for the *her7* knockdown). These residual oscillations were also present in anterior PSM, but no clear stripes of expression were formed, in agreement with experimental data. The simulations showed that *her1* and *her7,* with the biochemical parameters used, have essentially symmetrical roles. (This is because many parameters for *her7* were chosen to be the same as that of their *her1* counterpart, to make the computational study tractable.) Some asymmetry has been reported based on knockdown phenotypes [2]; for example, *her1* knockdown leads to some *her1* expression in anterior PSM, while *her7* knockdown leads to loss of expression of *her7* in anterior PSM. It is possible that some of this asymmetry stems from differences in mRNA in situ hybridization detection thresholds, which are unknown (*her7* could have a higher detection threshold than *her1*).

Simulated knockdown of *her1* and *her7* together showed upregulated expression in posterior PSM and a salt-and-pepper pattern in anterior PSM (Figure 5C and Video S6). This is consistent with experimental results showing generalized upregulation [5]. There was a slight discrepancy in that salt-and-pepper expression has been reported to occur throughout the PSM rather than specifically in anterior PSM [5]; this remains to be investigated. Strikingly, individual cells oscillated in the anterior PSM, even though the global pattern appeared constant. This brings computational confirmation to the hypothesis that a salt-and-pepper pattern can occur by desynchronization of cells, rather than by blocking of oscillations at different phases of the cycle in different cells [11]. In the double morphant, the clock started oscillating around the transition between posterior and anterior PSM. Because the oscillation period was roughly similar, the number of cycles experienced by cells in the PSM was about 50% lower than normal.

Another way in which the spatiotemporal oscillatory pattern can be disrupted is by grafting FGF-8 coated beads. This leads to minimal disruptions of oscillations when the bead is adjacent to posterior PSM, but to anterior extension of waves in the anterior PSM [31]. Such grafting experiments were simulated by assuming that an ectopic stripe of high *her13.2* mRNA expression is induced around the bead (Figure 6 and Video S7). When the bead was adjacent to posterior PSM, oscillations were not affected because the bead was assumed not to further increase *her13.2* expression. When the bead reached anterior PSM, posterior oscillations were still unaffected, but anterior oscillations, if imaged at the right phase, could show anterior extension of a wave in anterior PSM.

## Discussion

Direct characterizations of molecular interactions in the zebrafish somitogenesis clock network are scarce. Many dimerization combinations remain untested, and potential binding sites on the *her1*–*her7* and *deltaC* promoters remain unmapped. It would be a daunting task to test all possible molecular interactions and measure all biochemical reaction rates; the theoretical work presented here makes readily testable predictions and identifies select biochemical parameters of the network that are likely to have great impact on its behavior.

The existence of Her1–Her7 heterodimerization is a key feature to explain *her1* and *her7* morpholino knockdown phenotypes, with Her1–Her7 dimers having a protective role by competing with other dimers that repress transcription more strongly. The half-life of the Her1–Her7 heterodimer is predicted to have an important influence on the period of oscillation. Interestingly, dimerization of clock proteins is a feature shared with mouse and chick somitogenesis clocks [32]. However, the mechanism in those two clocks seems to be very different in that Lunatic fringe—which potentiates Notch activation by its ligand Delta—oscillates along with other clock genes, and that a positive feedback loop is likely to drive the oscillations [24].

The models studied here were kept simple to make it easier to extract essential features. The cellular aspects could be expanded by taking into account cell cycling throughout the PSM [29], blocking of mRNA transcription and protein translation in the mitotic phase of the cell cycle, possible cell mingling (observed in chicken [33,34]), and the effect of coupling on a 2-D or 3-D set of oscillators (rather than on a linear chain as in this study). It was assumed that the somitogenesis clock is already active in PSM progenitors (this has been suggested for early oscillations in chick [35], but does not seem to have been addressed in zebrafish); it would also be possible to have the clock inactive in progenitors and kick-started when cells join the posterior PSM. The molecular networks could also be expanded by having different enhancers active in posterior and anterior PSM, as shown experimentally [2]. This study shows that the presence of such different enhancers is not a fundamental requirement of the somitogenesis clock, but it might allow finer reproduction of the spatiotemporal pattern of gene expression and of its disruption by morpholino knockdown. Such an extension of the model might also allow for a role of *fused somites,* a gene essential for somite formation [36], whose activity is required for the propagation of expression waves into anterior PSM [6,10,37].

This study addressed the molecular mechanism of the somitogenesis clock oscillations. The mechanism by which the oscillations are read out to control mesoderm segmentation is not fully understood and is likely to be linked to the complex process of somite polarity establishment, most thoroughly studied in mouse [38]. Interestingly, out-of-phase oscillation of dimerization partners has been proposed as a mechanism to establish somite polarity [39]; however, the proteins considered in the present model do not oscillate out of phase. A model for segmentation based on reaction–diffusion of factors promoting anterior or posterior somite fate has also been proposed [40], but the genes considered in the present model cannot be related to such factors in a straightforward fashion.

A “clock and wavefront” model, first proposed by Cooke and Zeeman [41], is most often invoked to explain segmentation. However, modifications of the original model [30,42] suppose that clock and FGF-8 wavefront are independent, which has previously been shown not to be the case ([31]; see also Figure 4I in [30]). The present study details a molecular mechanism with strong experimental support by which FGF-8 interacts with the clock to regulate the spatiotemporal pattern of oscillation. This will in turn make it possible to investigate how clock and wavefront interact to regulate segmentation.

## Materials and Methods

The step function *S* used to shape the *her13.2* gradient is defined by

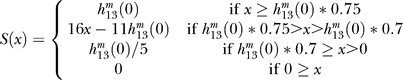



### Definition of suitable oscillations.

Deterministic oscillations were considered suitable if each interpeak distance in the course of the simulation fell between 10 min and 100 min, and if the amplitude of the peaks was sufficiently high, both in relative terms (at least a 5-fold difference between minimal and maximal values) and absolute terms (at least 30 molecules at the peak value). Potentially spurious peaks arising within 10 min after a previous peak were discarded from the analysis. Both *her1* and *her7* mRNA oscillations were assayed, each was required to meet these criteria, and the number of peaks undergone by each could not differ by more than one (so as to ensure roughly similar oscillation periods). Only *her1* mRNA period oscillation was measured (*her1* and *her7* play symmetrical roles in the models studied here), either as the distance between the last two peaks in a simulation run, so as to allow the system to have likely reached a limit cycle after the zero initial conditions used to start the simulation, or as the average of all distances between consecutive peaks in the simulation.

The algorithm above requires the absence of monotonicity changes between major peaks. As a control, a different algorithm was also used: the autocorrelations of the *her1* mRNA copy numbers through time were computed for increasing timeshifts starting from zero, and the period considered to be the first nonzero timeshift that produced a local maximum of the autocorrelation value. For deterministic simulations, the results were very close to that of the algorithm described above.

### Numerical simulations.

Deterministic simulations were performed with an adaptive-stepsize, 4th-order Runge–Kutta–Fehlberg algorithm [43] implemented in a custom C++ program (available on request). Numerical accuracy (taking into account both absolute and relative accuracies [43]) was set to 1%. Time points where derivatives are discontinuous (because of delays or because of the introduction or removal of oscillators in a chain of coupled oscillators) were forced to be part of the integration mesh. To speed up computations and ease RAM requirements, a subset of past solution values was stored, and delayed values required by the derivative function were linearly interpolated. To ascertain the accuracy of this method, a subset of results were compared with that obtained with a method providing 4th-order interpolation [44], with storage of all past integration steps; no significant difference was observed.

Stochastic simulations were implemented following the Gibson–Bruck algorithm [45], with a custom C++ program (available on request).

Simulations were carried out on a set of PowerPC G5 iMacs, PowerPC G5 PowerMacs, and Intel Core Duo iMacs (totaling about 16 processors), using GNU gcc 4.0.1 (with optimization setting on fast) and Intel icpc 9.1 as compilers, and on two SGI ALTIX 350 servers comprising a total of 32 Intel Itanium 2 processors and 64 GB of RAM, using gcc 3.2.3 (with optimization setting on fast) or icc 8.0 as compilers (the ALTIX servers being essentially used for stochastic simulations of chains of oscillators).

## Supporting Information

Video S1 Deterministic Simulation of a Chain of Oscillators Corresponding to the Model Sketched in Figure 1 (See Table 1 for Values of Parameters) Waves of expression spread from posterior to anterior PSM, as observed experimentally. To test the effect of oscillator-coupling through DeltaC-Notch signaling, a perturbation was induced by delaying a number of oscillators in the chain by 5 min, 50 min after the start of the simulation. After 1.3 h, the resulting perturbations in the spatial pattern were reduced.
(2.5 MB MOV)

Video S2 Stochastic Simulation of a Chain of Oscillators Corresponding to the Model Sketched in Figure 1, with Normal Coupling through Delta-Notch Signaling (1.9 MB MOV)

Video S3 Stochastic Simulation of a Chain of Oscillators Corresponding to the Model Sketched in Figure 1, with Disrupted Coupling Some cells were noticeably out of synchrony with their neighbors, and local synchrony was not as strong.
(1.9 MB MOV)

Video S4 Simulated Translation Knockdown of *her1*
Residual oscillations of diminished relative amplitude were observed, and stripe formation in anterior PSM was disrupted.
(795 KB MOV)

Video S5 Simulated Translation Knockdown of *her7*
Residual oscillations of diminished relative amplitude were observed, and stripe formation in anterior PSM was disrupted.
(823 KB MOV)

Video S6 Simulated Translation Knockdown of *her1* and *her7*
*her1* and *her7* were upregulated and did not oscillate in posterior PSM, but did oscillate in a salt-and-pepper pattern in anterior PSM.
(1.0 MB MOV)

Video S7 Simulated Grafting of an FGF-8 Coated Bead Oscillations in the region of ectopic *her13.2* expression were abnormal.
(2.5 MB MOV)

## Abbreviation

PSM: presomitic mesoderm
